# Investigating the potential association between the continuous-time random-walk MR diffusion model and lymph node metastasis and vascular invasion in patients with rectal cancer: A preliminary exploratory study

**DOI:** 10.1371/journal.pone.0361487

**Published:** 2026-10-30

**Authors:** Xiong Wu, Weijun Situ, Zhichao Feng, Haitao Yang, Cheng Yu, Qilin Yu, Junjiao Hu

**Affiliations:** 1 Department of Radiology, The Second Xiangya Hospital of Central South University, Changsha, Hunan, People’s Republic of China; 2 Central Research Institute, United Imaging Healthcare, Shanghai, People’s Republic of China; 3 The Key Laboratory of Biomedical Information Engineering of Ministry of Education, Institute of Health and Rehabilitation Science, School of Life Science and Technology, Xi’an Jiaotong University, Xi’an, Shaanxi, People’s Republic of China; University of Pisa, ITALY

## Abstract

**Objectives:**

To explore, as a preliminary study, the quantitative parameters of single-index and continuous-time random-walk (CTRW) magnetic resonance (MR) diffusion models and their association with lymph node metastasis, vascular invasion, and some prognostic factors in patients with rectal cancer.

**Methods:**

This retrospective, exploratory study included a total of 43 patients who were pathologically diagnosed with rectal cancer and underwent preoperative MRI between February 2022 and November 2023. The data were fitted using the single-index and CTRW diffusion models to derive ADC, Dm, α, and β parameters. Differences in quantitative diffusion parameters were assessed for lymph node metastasis, vascular invasion, and p53 status using t-tests and rank-sum tests. The associations of quantitative diffusion parameters with lymph node metastasis and vascular invasion were evaluated using receiver operating characteristic (ROC) analysis.

**Results:**

Lymph node metastasis was positive in 25 patients and negative in 18 patients; vascular invasion was positive in 30 patients and negative in 13 patients. In this initial investigation, the Dm and β values derived from the CTRW model showed a certain association with lymph node metastasis (AUCs of 0.76 and 0.76, respectively). Regarding vascular invasion, only the Dm value showed a certain association (AUC of 0.76), while the β value did not demonstrate a significant association after adjustment.The combination of Dm and β demonstrated stronger associations, with AUCs of 0.83 and 0.83, respectively.

**Conclusion:**

In this exploratory study, the diffusion parameters derived from the CTRW model may be associated with lymph node metastasis and vascular invasion in rectal cancer, but this requires external validation.

## Introduction

The guidelines of the National Comprehensive Carcinoma Network (NCCN) highlight the importance of neoadjuvant chemoradiotherapy for patients with rectal carcinoma [1]. The assessment of lymph node status plays a crucial role in determining appropriate treatment strategies, For lymph nodes with a short axis diameter equal to or greater than 5 mm, the size criterion is commonly used for distinguishing benign and malignant lymph nodes, yielding considerable accuracy [2]. Although some morphological features, such as boundary contours, signal intensity, internal structure, and chemical shift effects, have contributed to improved diagnostic accuracy in assessing lymph node status, their utility is still influenced by the subjective judgments of different radiologists [2–4]. Morphological criteria are valuable in clinical diagnosis, but researchers have shown interest in the various quantitative parameters offered by functional magnetic resonance imaging (MRI), as they provide additional information about the microenvironment beyond morphological features. Diffusion-weighted imaging (DWI) enables quantification of the Brownian motion of water molecules in both the intracellular and the extracellular compartments without the need for contrast medium administration. The apparent diffusion coefficient (ADC), a quantitative parameter derived from DWI, is a quantitative parameter derived from DWI that offers insights into the microstructure of the cellular microenvironment on the basis of the Gaussian behavior of water diffusion in tissues [5].

With the advancement of functional MRI techniques, research on rectal carcinoma imaging has shifted from macroscopic to microscopic and from gross morphology to molecular biology. Various DWI models have gained significant attention in the investigation of rectal carcinoma. Conventional DWI based on a monoexponential model quantitatively assesses the degree of water molecule diffusion restriction, thereby reflecting the pathological and physiological alterations in the tissue microstructure. However, the monoexponential model assumes a Gaussian distribution of water molecules and overlooks the intricate tumor microenvironment. Consequently, the ADC value often overestimates the weight of water molecule diffusion and fails to accurately reflect the true diffusion motion within tumor tissues. One study demonstrated the potential of the ADC in identifying the lymph node status in rectal carcinoma patients [6,7]. In contrast, another study reported that although DWI is helpful for the detection of lymph nodes, the ADC cannot reliably differentiate between malignant and benign lymph nodes [8]. The inconsistencies in these findings may be attributed to the complexity and nonuniformity of the tissue organization, which leads to water diffusion deviating from the Gaussian distribution, limiting the effectiveness of the ADC [9].

Therefore, non-Gaussian diffusion models have emerged to address current needs. The non-Gaussian CTRW model, which is based on multiple b-value DWI images, introduces novel quantitative parameters for characterizing nonGaussian diffusion in heterogeneous media. Specifically, the CTRW model recognizes that water molecules undergo variable displacements (water jumps) or require variable times (water capture) for movement. These two situations correspond to the heterogeneity of temporal and spatial diffusion, described by parameters α and β, respectively. These two new parameters, in conjunction with the anomalous diffusion coefficient Dm, enable the CTRW model to provide a multidimensional evaluation of tissue changes [10].

Thus, the purpose of this study was to preliminarily explore the quantitative DWI parameters derived from the monoexponential model and advanced CTRW model and their potential associations with lymph node metastasis and vascular invasion in rectal carcinoma patients, as well as to preliminarily investigate the possible correlations between these parameters and prognostic factors.

## Materials and methods

### Patients

This prospective study was approved by the Ethics Committee of the Second Xiangya Hospital, Central South University (NO. S2021SFYLJS0082). All participants gave their written informed consent. Patients with pathologically confirmed rectal carcinoma via biopsy who underwent pelvic MRI examinations were enrolled from February 2022 to November 2023. The inclusion criteria were as follows: (1) patients with rectal carcinoma scheduled for total mesorectal excision; (2) no prior radiotherapy or chemotherapy before surgery; and (3) available preoperative MRI data. The exclusion criteria were as follows: (1) failure to undergo surgical treatment at our hospital; (2) histological type other than adenocarcinoma; (3) poor image quality affecting lesion segmentation; and (4) a history of other malignant tumors. The flow chart for the selection of participants for this study is shown in Fig 1.

**Fig 1:**
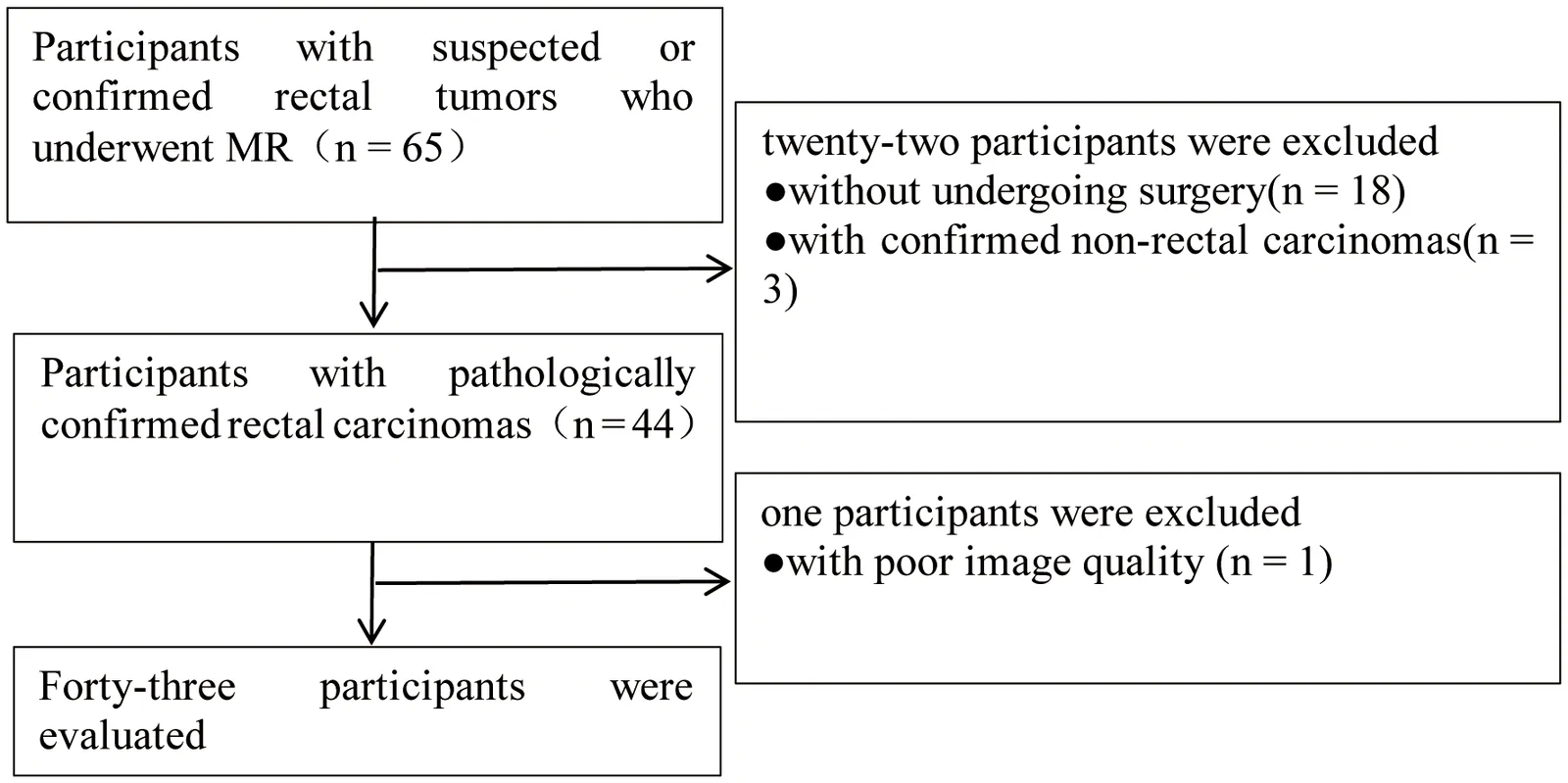
Flowchart of the study population.

### MRI examinations

MRI was performed with a 3.0 T system (uMR790, United Imaging Healthcare, Shanghai, China) with 12-channel phased-array body coils. Patients were instructed to fast and consume magnesium sulfate to prepare their intestines accordingly before the examination. Straps were used to secure the lower abdomen to reduce respiratory artifacts from lower abdominal movement. The axial multi b-value protocol of DWI and CTRW was as follows: b values = 0 (1), 50 (1), 80 (1), 100 (1), 150 (1), 200, 300 (2), 500 (2), 800 (2), 1000 (4), 1300 (4), 1700 (6), 1800 (6), 2400 (6), 3000 (8), and 3600 (10) s/mm^2^. The number of averages is presented in parentheses. Other sequences included T2-weighted imaging (T2WI) in the sagittal position and oblique axial position perpendicular to the long axis of the tumor; unenhanced T1-weighted imaging (T1WI) in the oblique axial position; and dynamic T1WI in the oblique axial, sagittal, and coronal positions. The detailed parameters of these sequences are summarized in Table 1.

**Table 1:** MRI protocols.

MRI Protocol	TR (ms)	TE (ms)	Slice Thickness (mm)	slice	FoV	Matrix	Scanning time
Precontrast enhanced sequences							
Sagittal T2WI_FS_ACS	3573	140	4	23	240 × 240	384 × 384	1′16″
Sagittal T2WI_ACS	3312	140	4	23	240 × 240	384 × 384	1′08″
Oblique transverse CTRW	2560	80	4	20	400 × 400	120 × 120	7′14″
Oblique coronal T2WI_ ACS	3340	140	4	23	240 × 240	384 × 384	1′08″
Oblique transverse T2WI _FS_ACS	3622	130	4	26	240 × 240	388 × 432	1′28″
Oblique transverse T2WI _ACS	3340	130	4	26	240 × 240	388 × 432	1′20″
Oblique transverse T1WI_ ACS	460	8.7	4	26	240 × 240	430 × 480	1′18″
Transverse quick3D T1WI_FS_CS	3.8	1.6	1.1	144	250 × 300	259 × 312	23″
Postcontrast enhanced sequences							
Sagittal T1WI_FS	536	8.3	4	23	240 × 240	345 × 432	1′35″
Oblique coronal T1WI_ FS	514	7.7	4	23	240 × 240	345 × 432	1′30″
Transverse quick3D T1WI_FS_CS	3.8	1.6	1.1	144	250 × 300	259 × 312	23″

T1W, T1-weighted imaging; T2W, T2-weighted imaging; FS, fat suppression; ACS, AI-assisted compressed sensing; uCS, combined compressed sensing; TR, repetition time; TE, echo time; FOV, field of view; “means minutes;” means seconds.

### Image processing and analysis

The monoexponential model was calculated for comparison via the following equation:


$$\begin{array}{c} \frac{\text{S}}{{\text{S}}_{\text{0}}}={\text{e}}^{-\text{bADC}}, \end{array}$$

where S_0_ and S are the signal intensities in the voxel with b values of 0 and 800 mm^2^/sec, respectively [10].

The CTRW model was calculated through the following fitting formula:


$$\begin{array}{c} \frac{\text{S}}{{\text{S}}_{\text{0}}}={\text{E}}_{\alpha }[{-({\text{bD}}_{\text{m}})}^{\beta }], \end{array}$$

where S_0_ is the signal strength without diffusion weighting, Dm is the anomalous diffusion coefficient, and α is a parameter related to time diffusion heterogeneity, indicating the possibility of water molecules being trapped within the structure; β is a parameter related to spatial diffusion heterogeneity, indicating that water molecules can generate variable spatial displacement with each movement. Eα is a Mittag Lefler function of order α, which uses the iterative Levenberg Marquardt method in MATLAB (MathWorks, Natick, MA, USA) to generate CTRW parameter maps for Dm, α, and β, and b is the value of b [10, 11].

Two abdominal radiologists with 5–10 years of experience in rectal MRI, who were blinded to the patients’ clinical and pathological information, independently performed whole-tumor segmentation. ROIs were drawn on the oblique transverse T2WI images and then transferred to the corresponding DWI and CTRW parameter maps using image registration. Specifically, the entire tumor volume was segmented on all slices showing tumor, and tumor boundaries were defined based on combined T2WI and DWI signal characteristics. To address geometric distortion on DWI, DWI images were coregistered to the oblique transverse T2WI using affine transformation to correct for geometric distortion; residual misregistration was visually checked and manually corrected when necessary. Segmentation, registration, and parameter extraction were performed using open-source ITK-SNAP software (https://www.itk-snap.org) and MATLAB (MathWorks, Inc., Natick, MA). After coregistration, the ROIs were transferred to the corresponding ADC, DmDm, αα, and ββ maps, and the values were extracted. Before formal segmentation, both readers underwent calibration training on 5 cases to standardize the delineation approach. Disagreements were resolved by consensus discussion with a third senior radiologist (15 years of experience).

The Mittag-Leffler function fitting was performed on a voxel-wise basis using the iterative Levenberg-Marquardt method in MATLAB. Initial parameter estimates were set as Dm = 1.0 × 10^−3^ mm²/s, α = 0.8, and β = 0.6 based on preliminary fitting. The convergence criterion was a relative change of < 10^−6^. Voxels that failed to converge (defined as relative change ≥ 10^−6^ after 1000 iterations, approximately 4.7% of all voxels) were excluded from the analysis. ROI-level parameters were calculated as the median of all voxel-wise values within the ROI.

### Histopathological evaluation

The final histopathological analysis of tumor samples was used as the reference standard. All surgical specimens were stained with hematoxylin and eosin (HE) and underwent immunohistochemical staining for Ki-67, p53, and PD-L1. A specialized gastrointestinal pathologist classified the lymph node status as metastatic or non-metastatic under a microscope; a specimen was considered lymph node metastasis-positive if one or more of the excised lymph nodes showed metastatic involvement. The same criteria were applied to vascular invasion. It is worth noting that additional lymph nodes not detected on MRI images were also excised from the specimens and examined pathologically to ensure an adequate number of lymph nodes were available for pathological assessment [12].

### Statistical analysis

All the statistical analyses were performed with SPSS software (version 26; SPSS, Inc., Chicago, IL) and MedCalc software (version 18.2; MedCalc Software bvba, Ostend, Belgium). Interreader agreement for the measurements of the ADC, Dm, α, and β was assessed with intraclass correlation coefficients (ICCs). An ICC less than 0.20 indicates poor agreement; 0.21 to 0.40 indicates fair agreement; 0.41 to 0.60 indicates moderate agreement; 0.61 to 0.80 indicates good agreement; and 0.81 to 1.00 indicates excellent agreement. The Shapiro–Wilk test was used to evaluate the normality of the data distributions. Continuous variables are expressed as the mean ± standard deviation (SD) or the median with an interquartile range (IQR) on the basis of a normality test. α and β values followed a normal distribution (Shapiro-Wilk p > 0.05), while Dm and ADC values did not (Shapiro-Wilk p < 0.05), hence the different presentation formats (mean±SD vs. median[IQR]). Independent sample t tests or Mann‒Whitney U tests were used for comparisons between groups and corrected using the Benjamini-Hochberg method for multiple comparisons. A total of 12 comparisons were tested (4 parameters × 3 outcomes) with a false discovery rate threshold of 0.05 using the Benjamini-Hochberg method.Receiver operating characteristic (ROC) curves were constructed using pathological histology as the gold standard to evaluate the predictive performance of ADC and CTRW parameters for lymph node metastasis and vascular invasion in patients with rectal cancer. Subsequently, the combined performance of CTRW parameters was assessed using binary logistic regression in conjunction with ROC curve analysis. The area under the ROC curve (AUC), sensitivity, and specificity were calculated. The corresponding cutoff values yielding optimal diagnostic accuracy were determined using the maximum Youden index. AUCs were compared using the DeLong method. The AUC was validated using the leave-one-out cross-validation method to avoid overfitting.Leave-one-out cross-validation was performed by iteratively excluding one patient, fitting the logistic regression model on the remaining n-1 patients, and evaluating performance on the excluded patient. This process was repeated for all patients, and the average AUC across all folds is reported as the LOOCV-adjusted AUC. Feature selection and cut-off determination were performed within each training fold to avoid data leakage. A p-value of less than 0.05 was considered statistically significant.The study was not prospectively powered to detect clinically meaningful differences. The sample size was determined by convenience based on available cases during the study period.Post-hoc power analysis was performed using GPower 3.1. Given the sample sizes (LNM+: n = 18, LNM−: n = 25; VI+: n = 13, VI−: n = 30), the minimum detectable Cohen’s d (two-sided t-test, α = 0.05, power = 0.80) was 0.86 for LNM comparisons and 0.96 for VI comparisons. The events-per-variable ratio for the combined model was 12.5 for LNM but only 6.5 for VI, indicating inadequate power for reliable logistic regression in the VI analysis.

## Results

### Clinical and pathological characteristics

A total of 43 patients with rectal carcinoma were included. The clinical and pathological characteristics of the patients are shown in Table 2. Among them, 3 participants had moderately to highly differentiated, well-differentiated, and highly differentiated adenocarcinomas; 36 participants had moderately differentiated adenocarcinomas; and 4 patients had moderately to poorly differentiated adenocarcinomas. Twenty-five participants experienced perirectal lymph node metastasis, and 13 participants experienced vascular invasion. Fig 2 shows the conventional MR sequence, diffusion parameter sequence, and pathological section from a patient who had both lymph node metastasis and vascular invasion.

**Table 2:** Clinical and pathological characteristics of the patients.

	LNM(-)	LNM(+)	*p* value	VI(-)	VI(+)	*p* value
Number	25	18		30	13	
Age	60.2 ± 10.3	60.7 ± 11.8	0.890	60.3 ± 9.8	60.7 ± 13.2	0.931
Ki-67(%)	71.6 ± 18.1	70.6 ± 15.2	0.839	72.0 ± 17.1	69.2 ± 16.4	0.621
P53	(17 + /8-)	(15 + /3-)	0.270	(22 + /8-)	(10 + /3-)	0.810
type	Medium to high differentiation or above (3), Medium differentiation (36), Low to medium differentiation (4)					

The numbers in parentheses are the raw data of the P53 mutant and wild-type strains. *p* values refer to significant differences in clinical and pathological characteristics.

Ki-67, Ki-67 proliferation. P53, P53 gene mutation.

**Fig 2:**
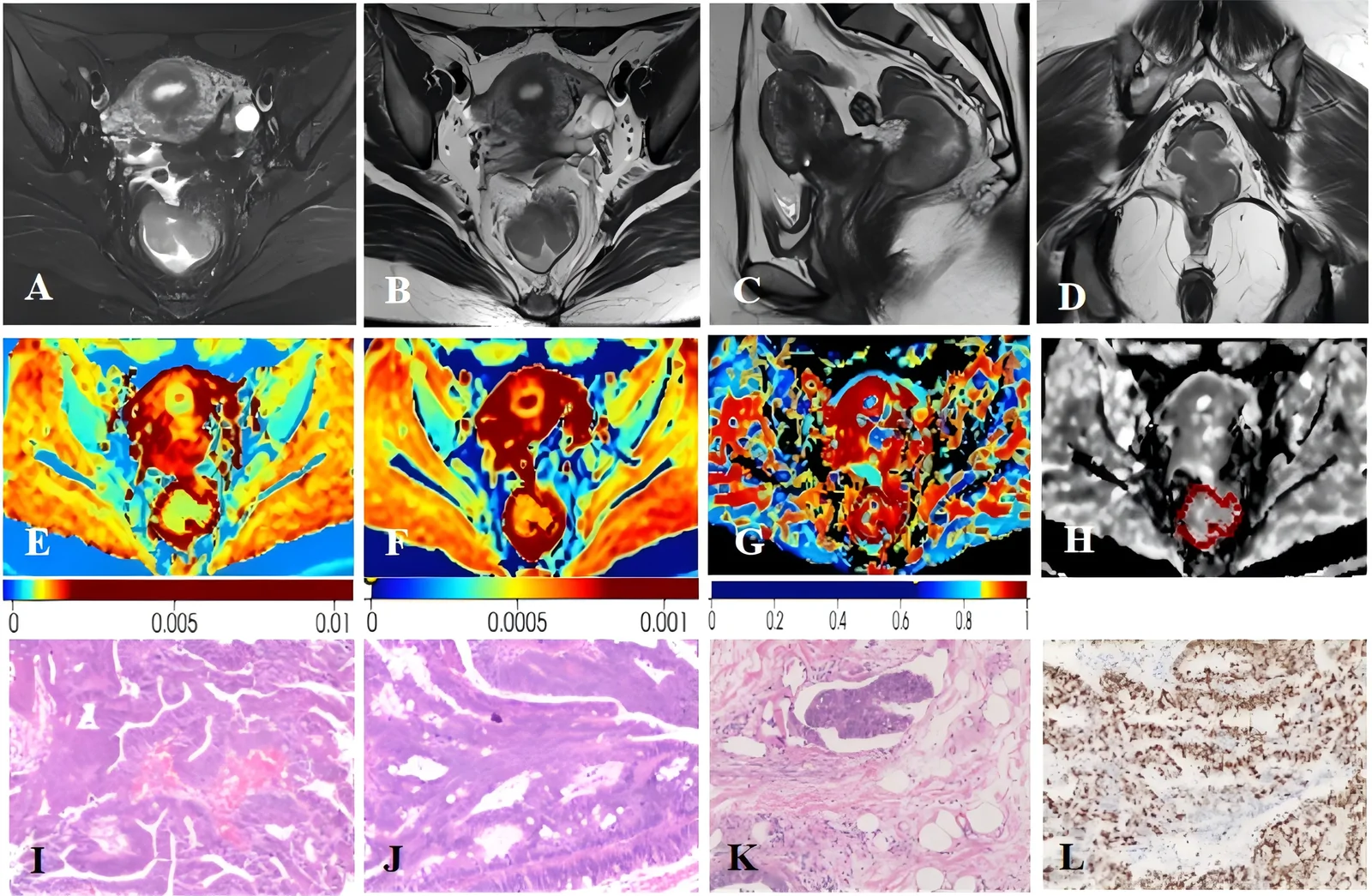
A case of rectal carcinoma with lymph node metastasis and vascular invasion using CTRW.

A 42-year-old female was diagnosed with moderately differentiated rectal adenocarcinoma.(A) to (D) are Oblique transverse T2WI FS ACS, Oblique transverse T2WI ACS, Sagittal T2WI ACS, and Oblique coronal T2WI ACS, respectively.(E) to (H) pseudo colorized images showed the αCTRW(E), βCTRW(F), DmCTRW(G), ADC(H) maps derived from the conventional DWI, continuous time random walk (CTRW) respectively. β and Dm maps show pseudo colorized images of high signal intensity in the lesion area. (I) and (J) are pathological sections (HE 100×) of the patient, and the pathological type was Medium differentiation adenocarcinomas.(K) suggested the patient pathological results VI (+).(L) is immunohistochemical staining, which indicated high expression of Ki-67 in tumors.

### Interobserver agreement of Dm, β, α, and ADC measurements

The ICCs with 95% confidence intervals (CIs) for the representative mean and median metrics of Dm, β, α, and ADC are shown in Table 3. In general, the mean metrics of Dm, β, α, and ADC all show excellent agreement (range of ICCs, 0.86–0.94).

**Table 3:** Interobserver agreement for the mean metrics of Dm, β, α, and ADC.

Parameters	ICC	95% CI	*p* value
Dm	0.86	0.75-0.92	**0.001**
β	0.89	0.73-0.95	**0.001**
α	0.94	0.89-0.97	**0.001**
ADC	0.92	0.85-0.96	**0.001**

ICC < 0.40, poor repeatability; ICC > 0.80, good repeatability.

Bold value indicates *p* < 0.05.

CI, confidence intervals; ICC, intraclass correlation coefficient.

### Summary of diffusion parameters

There was a statistically significant difference in the diffusion Dm value (1.07(0.84,1.21)×10^−3^ mm²/s vs. 0.84(0.71,0.90)×10^−3^ mm²/s, *p* = 0.004, adjusted *p* = 0.03) and β value (0.63 ± 0.05 vs. 0.57 ± 0.07, *p* = 0.005, adjusted *p* = 0.03) of the CTRW model between the positive and negative groups of intestinal lymph node metastasis. Regarding peripheral vascular invasion, there was a statistically significant difference in the Dm value (1.14(0.89,1.33)×10^−3^ mm²/s vs. 0.84(0.74,0.91)×10^−3^ mm²/s, *p* = 0.008, adjusted *p* = 0.032) between the positive and negative groups; however, the difference in the β value did not remain statistically significant after adjustment (adjusted *p* = 0.08). No statistically significant differences were detected in the alpha values (*p* = 0.710, *p* = 0.329) or apparent diffusion coefficient (ADC) values (*p* = 0.133, *p* = 0.091) of the DWI model between the groups with positive and negative perirectal lymph node metastasis and vascular invasion. The detailed diffusion parameters are listed in Table 4.

**Table 4:** Descriptive statistics of the diffusion parameter values for LNM and VI.

Diffusion parameter	LNM(-)	LNM(+)	*p* value	VI(-)	VI(+)	*p* value
ADC(×10^−3^ mm²/s)	0.57 (0.55,0.66)	0.65 (0.57,0.74)	0.228(0.133)	0.58 (0.56,0.66)	0.68 (0.57,0.76)	0.182(0.091)
Dm(×10^−3^ mm²/s)	0.84 (0.71,0.90)	1.07 (0.84,1.21)	**0.030(0.004)**	0.84 (0.74,0.91)	1.14 (0.89,1.33)	**0.032(0.008)**
α	0.79 ± 0.10	0.80 ± 0.09	0.852(0.710)	0.79 ± 0.09	0.82 ± 0.09	0.494(0.329)
β	0.57 ± 0.07	0.63 ± 0.05	**0.030(0.005)**	0.58 ± 0.07	0.62 ± 0.05	0.082(0.034)

Bold value indicates *p* < 0.05.

The unadjusted P values are presented in parentheses.

LNM, lymph node metastasis; VI, vascular invasion.

### Evaluation of the performance of diffusion indicators in assessing peritoneal LNM and VI

Table 5 (Fig 3) shows the performance of Dm, β, and the combined parameter. Among the independent parameters evaluated for lymph node metastasis, Dm had the highest specificity (96%) with a cutoff value of 0.96 × 10^−3^ mm²/s, while the β parameter had the highest sensitivity (88.9%) with a cutoff value of 0.56. There was no significant difference in AUC values between Dm and β (0.76 vs. 0.76; LOOCV-adjusted AUC = 0.76 vs. 0.73, *p* = 0.97). Furthermore, among the parameters associated with peripheral vascular involvement, Dm had higher specificity (93.3%) with a cutoff value of 1.07 × 10^−3^ mm²/s, while the β parameter had higher sensitivity (84.6%) with a cutoff value of 0.58. There was also no significant difference in the AUC values of these two parameters (0.76 vs. 0.71; LOOCV-adjusted AUC = 0.75 vs. 0.72, *p* = 0.713). Whether used to diagnose lymph node metastasis or vascular invasion, the combination of Dm and β yielded higher AUC values (AUC = 0.83, LOOCV-adjusted AUC = 0.83, *p* < 0.001; AUC = 0.83, LOOCV-adjusted AUC = 0.79, *p* = 0.002). It should be noted that the sensitivity and specificity estimates are based on a small number of positive cases (n = 13 for VI), making them potentially unstable. The specificity of 93.3% for Dm in VI detection is based on only 30 negative cases.

**Table 5:** Results of the ROC analysis of Dm, β, α, and the ADC alone or in combination for the discrimination of LNM and VI.

	LNM			VI		
	Dm	β	Dm,β	Dm	β	Dm,β
Cut-off	0.95	0.56	0.35	1.07	0.58	0.41
Sensitivity^*^	61.1	88.9	83.3	61.5	84.6	69.2
Specificity^*^	96.0	60.0	72.0	93.3	60.0	83.3
Accuracy^*^	75.7	76.8	78.6	71.1	77.2	73.5
AUC(95%CI)	0.76 (0.60, 0.92)	0.76 (0.61, 0.90)	0.83 (0.71, 0.95)	0.76 (0.57, 0.94)	0.71 (0.54, 0.87)	0.83 (0.70, 0.96)
*p* value	**0.004**	**0.005**	**0.001**	**0.008**	**0.034**	**0.001**
Youden’s index	0.58	0.49	0.55	0.48	0.45	0.53

*Numbers are percentages, and numbers in parentheses are the 95% confidence intervals of the AUC values. The unit of the Dm cut-off value is × 10^–3^ mm²/s.

Bold value indicates *p* < 0.05.

AUC, area under the receiver operating characteristic curve.

**Fig 3:**
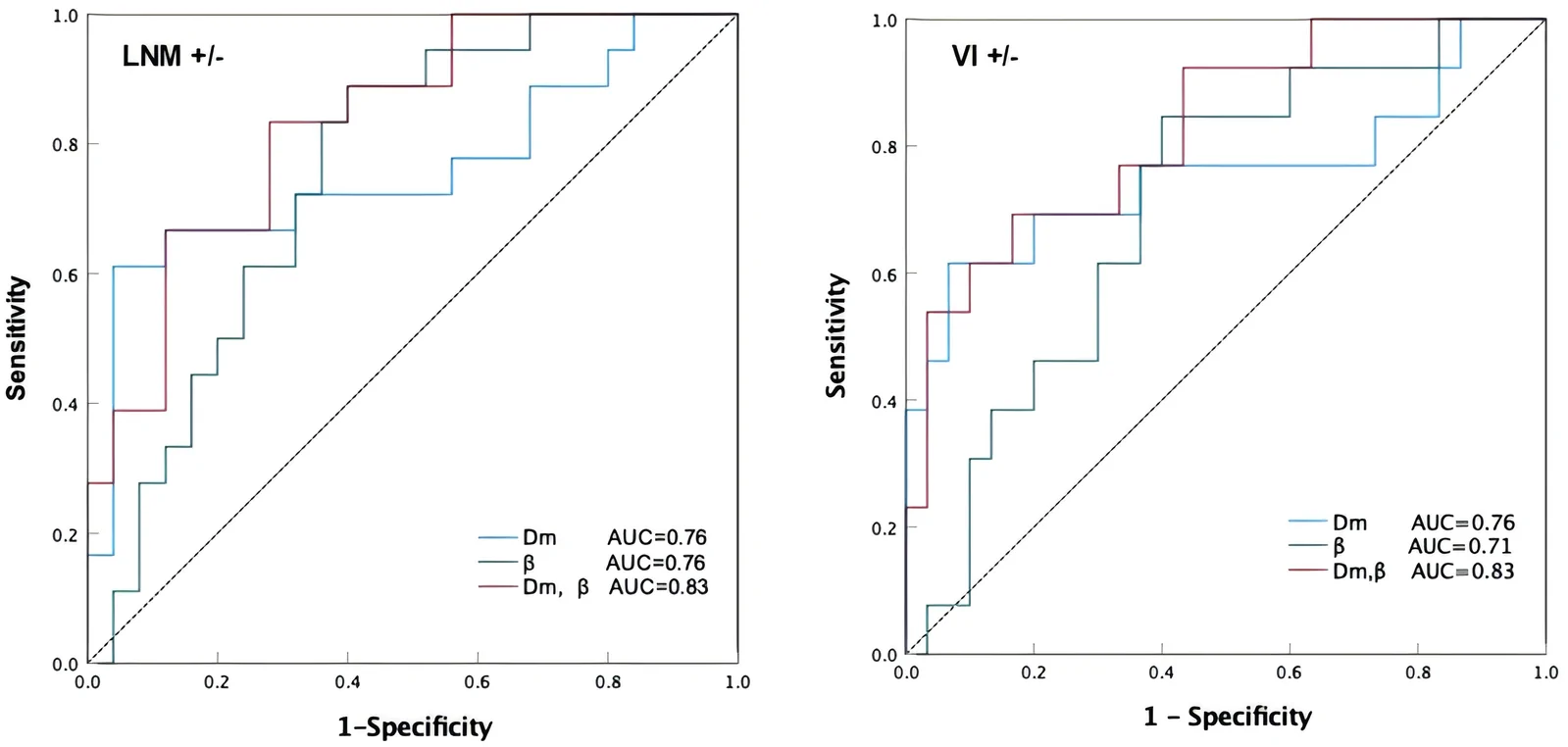
The ROC curves of diffusion parameter in distinguishing between LNM+/LNM- and VI+/VI-groups. ROC, receiver operating characteristic; LNM, lymph node metastasis; VI, vascular invasion.

### The preliminary exploration of the association between diffusion parameters and immunohistochemical markers in patient lesions

Exploratory analysis of the association between CTRW parameters and p53 status was performed and is presented in S1 Fig. Due to the small number of p53 wild-type cases (n = 3), these results should be interpreted with extreme caution.

## Discussion

Numerous studies on various types of carcinoma have demonstrated a significant negative correlation between diffusion coefficients from different diffusion models, such as the conventional ADC and the mean diffusion rate (MD) of the DKI model, and tissue cell density. This phenomenon is believed to reflect the restricted water diffusion associated with increased cell density [7,13]. Current studies consistently indicate that the diffusion coefficient Dm derived from the CTRW model is significantly lower for malignant tumors and can be used for the differential diagnosis of benign and malignant tumors, including breast carcinoma and bladder carcinoma [14–16]. However, no study to date has investigated the relationship between CTRW parameters of solid tumors and lymph node metastasis or vascular invasion in patients with rectal cancer. In the present study, ADC based on conventional DWI could not effectively distinguish between benign and malignant lymph nodes, nor could it predict vascular invasion status, a finding consistent with previous studies [8]. Dynamic contrast-enhanced MRI (DCE-MRI) has been used to quantitatively and qualitatively evaluate metastatic lymph nodes and the vascular microenvironment. However, owing to the necessity of Gd-DTPA injection, adverse events are unavoidable in clinical applications, and it is contraindicated for patients with renal dysfunction, with severe cases even leading to nephrogenic systemic fibrosis [17,18]. In this study, we preliminarily explored the connections between key parameters of the CTRW model and lymph node metastasis as well as vascular invasion. These findings provide certain reference value for the future noninvasive assessment of lymph node metastasis and vascular invasion in rectal cancer using the CTRW model.

In this study, the continuous-time random walk (CTRW) diffusion model was used to preliminarily explore its potential associations with the occurrence of lymph node metastasis and vascular invasion in rectal carcinoma patients. The tumor parameters Dm and β, which are derived from the CTRW model, were significantly greater in patients with positive lymph node metastasis or vascular invasion than in those with negative pathological outcomes. In patients with lymph node metastasis and vascular invasion, higher Dm values were observed, a finding that may contradict conventional expectations. Potential explanations for this observation are as follows.The observed higher Dm values in patients with lymph node metastasis and vascular invasion may reflect differences in tumor microstructure, such as altered cellular density, extracellular space, or the presence of necrotic or mucinous components. However, these interpretations are speculative, as our study did not include histopathological quantification of cellularity, necrosis, collagen content, or MMP expression. Future studies incorporating correlative histopathological analysis are needed to elucidate the biological basis of these diffusion parameter associations.Moreover, when Dm and β were combined, the resulting area under the curve (AUC) values were slightly greater than those obtained when either parameter was used independently. The imbalance in sample sizes between the vascular invasion‑positive and vascular invasion‑negative groups may have introduced bias into the calculation of sensitivity and specificity, leading to reduced stability of the results. Therefore, the reliability of these findings needs to be further validated with larger sample sizes in future studies.In addition, the β parameter showed no significant association with p53 status in colorectal cancer patients following internal adjustment.

Evaluating the prognostic factors for rectal carcinoma patients holds significant clinical importance. The correlation between the ADC and immunohistochemical markers has been a subject of considerable debate, with varying findings across different studies [19]. Some research results have indicated a significant negative correlation between the apparent diffusion coefficient (ADC) and Ki-67, a proliferation index of tumor cells, as an increase in Ki-67 implies increased cell number and density [20,21]. However, other studies have reported no significant difference in the apparent diffusion coefficient (ADC) between high- and low-Ki-67 groups [22]. In our study, no connection was observed between the ADC and immunohistochemistry indices, which was consistent with previous findings [22,23]. Recently, histogram analysis has been employed to evaluate different diffusion parameters in carcinoma research. For example, histogram analysis of ADC values within the entire tumor volume has shown inconsistent results, with parameters related to immunohistochemical indicators being the 10th percentile ADC and the 95th percentile ADC, respectively [22,24]. Usually, histogram analysis is used to mitigate the influence of other components, such as necrosis in the region of interest (ROI), on the average diffusion coefficient. In this study, this potential impact was reduced by carefully selecting the ROI.

P53 is one of the most common mutated genes in colorectal carcinoma, with a high mutation rate in rectal carcinoma [25]. In addition to the Ki-67 index, the P53 status in rectal carcinoma is also a prognostic factor [26]. Previous studies have shown that the signal intensity (SImean) of ammoniated proton transfer (APT) imaging is related to the expression level of p53. APT imaging can serve as a noninvasive biomarker for evaluating genetic prognostic factors in rectal adenocarcinoma patients [27]. Similarly, in this study, we preliminarily explored the connection between parameter β of the CTRW model and p53. Although this correlation did not pass the internal validation of leave-one-out cross-validation. Future studies may attempt to increase the sample size and adopt more robust statistical methods to further investigate this relationship.

With the further development of diffusion models and under the assumption of nonGaussian diffusion behavior of water molecules, the discriminative ability of advanced models such as the CTRW model has been extended to the internal heterogeneity of tissues [28]. Therefore, it can provide more comprehensive information on tissue characteristics in terms of cellular structure and heterogeneity. In addition to Dm, the derived parameters α and β in the CTRW model represent the temporal and spatial heterogeneity of the tissue, respectively [15]. An increase in the β value may be associated with lymph node metastasis and vascular invasion in patients with rectal cancer. Of note, compared with a single diffusion parameter, the combined use of derived parameters (Dm and β) can further enhance the ability to investigate lymph node metastasis and vascular invasion status in rectal cancer.

In the pathological evaluation of solid tumors, vascular invasion (VI) is defined as the presence of tumor cell clusters within the lumen of blood vessels (veins or arteries). In many studies, vascular invasion (VI) serves as an umbrella term encompassing two major categories: lymphovascular invasion (LVI) and blood vascular invasion (BVI, i.e., venous/arterial invasion). Since lymphatic vessels and blood vessels are often difficult to distinguish on pathological sections, many pathology reports use the collective term “vessel invasion” (or “vascular invasion” as a general term for both) [29,30]. For a long time, it has been regarded as an independent predictor of tumor recurrence and metastasis. Research has shown a strong association between VI and liver and lung metastases of tumors [31]. In addition to other pathological and molecular features, such as poorly differentiated tumors, tumor perforation, perineural invasion, and microsatellite instability, VI has been identified as an unfavourable prognostic factor. The European Society for Medical Oncology (ESMO) guidelines consider positive magnetic resonance-detected vascular invasion (mrVI) a high-risk factor for rectal cancer patients. The status of mrVI has become an indication for neoadjuvant therapy [32]. Therefore, VI has emerged as a crucial factor in treatment decision-making processes for rectal cancer patients. However, the sensitivity and specificity of conventional MRI sequences for detecting vascular invasion are 68.2% and 93.2%, respectively [33]. Based on this, we preliminarily explored the association between quantitative parameters derived from the CTRW model and perirectal lymph node metastasis as well as vascular invasion in rectal cancer. Under the same conditions, the ADC parameter from the DWI model did not show a significant association, representing an exploratory finding that warrants further investigation. These preliminary results provide a hypothesis-generating basis for future research, though direct comparative studies are needed to assess the relative utility of the CTRW versus conventional DWI models. Our study did not directly compare CTRW performance to conventional morphological MRI assessment or radiologist assessment of lymph node status. The added value of CTRW parameters over existing clinical and imaging assessment remains unknown.

This study has several limitations. First, due to the limited number of patients, this single-center study may introduce bias, and in the absence of external validation, the findings are preliminary and exploratory. Additionally, although most patients were pathologically classified as having moderately differentiated adenocarcinoma, more than 20% of patients did not fall into this category. This may have led to differences in diffusion parameters between patients with different pathological grades. Furthermore, all patients underwent radical resection for rectal cancer, and patients with locally advanced disease who had received neoadjuvant therapy were excluded, which may have introduced validation bias and limited the generalizability of the findings to the broader rectal cancer population. Finally, theoretically, low differentiation is associated with lymph node metastasis. However, in this study, only two-thirds of patients with moderately to poorly differentiated tumors had lymph node metastasis, which may be due to our small sample size. In addition, the cutoff values (e.g., Dm > 1.07 × 10^−3^ mm²/s) and performance metrics are derived from a single small cohort and cannot be generalized. Independent validation in a larger cohort is essential before any clinical application. As a single-center study, our findings reflect the characteristics of a single institutional population. MRI protocols, parameter fitting approaches, and pathological assessment may differ elsewhere. No external validation cohort was used to test the generalizability of the proposed associations. LOOCV,while useful for internal validation, cannot replace true external validation in an independent cohort. Therefore, the generalizability of our findings is substantially limited. Another limitation is that interobserver agreement was assessed only at the ROI level, and slice-level ICCs were not calculated. In addition, whole-tumor summary metrics may obscure intratumoral regional heterogeneity relevant to LNM and VI. Future studies should include per-slice ICC analysis and voxel-wise, histogram-based, or spatial subregion analyses.

## Conclusion

This study preliminarily investigated the association between the CTRW model parameters Dm and β and lymph node metastasis and vascular invasion in patients with colorectal cancer. Due to the small sample size and single-center design, the reliability of the association between the quantitative diffusion parameters in the CTRW model and prognostic factors remains limited. Furthermore, due to the lack of an external validation cohort, only LOOCV was performed as the sole internal validation method; therefore, the results are preliminary and exploratory. The study included only patients who underwent radical resection for rectal cancer, excluding those with locally advanced disease who received neoadjuvant therapy, which may introduce validation bias and limit the generalizability of the findings to the entire rectal cancer population. Nevertheless, these findings provide some valuable insights. Future studies should increase the sample size and refine statistical methods to enhance the study’s validity.

## Supporting information

S1 Fig Exploratory analysis of the association between β parameter and p53 status. (DOCX)

S1 Table All Patients Data. https://example.com/supplementary-table-all-patients-data.
(XLSX)

## References

[1] Benson AB, Venook AP, Adam M, Chang G, Chen YJ, Ciombor KK. NCCN Guidelines® Insights: Rectal Cancer, Version 3.2024. J Natl Compr Canc Netw. 2024;22(6):366–75. doi: 10.6004/jnccn.2024.0041 39151454

[2] Doyon F, Attenberger UI, Dinter DJ, Schoenberg SO, Post S, Kienle P. Clinical relevance of morphologic MRI criteria for the assessment of lymph nodes in patients with rectal cancer. Int J Colorectal Dis. 2015;30(11):1541–6. doi: 10.1007/s00384-015-2339-y 26260478

[3] Yuan Y, Pu H, Chen G-W, Chen X-L, Liu Y-S, Liu H, et al. Diffusion-weighted MR volume and apparent diffusion coefficient for discriminating lymph node metastases and good response after chemoradiation therapy in locally advanced rectal cancer. Eur Radiol. 2021;31(1):200–11. doi: 10.1007/s00330-020-07101-3 32740816

[4] Maatman IT, Maas MC, Ypma S, Block TK, van den Elshout R, Veltien A, et al. High-resolution free-breathing chemical-shift-encoded MRI for characterizing lymph nodes in the upper abdomen. Invest Radiol. 2025;60(7):434–43. doi: 10.1097/RLI.0000000000001147 39761203

[5] Yang ZL, Li Y, Zhan CA, Hu YQ, Guo YH, Xia LM, et al. Evaluation of suspicious breast lesions with diffusion kurtosis MR imaging and connection with prognostic factors. Eur J Radiol. 2021;145:110014. doi: 10.1016/j.ejrad.2021.110014 34749223

[6] Hao Y, Zheng J, Li W, Zhao W, Zheng J, Wang H, et al. Ultra-high b-value DWI in rectal cancer: image quality assessment and regional lymph node prediction based on radiomics. Eur Radiol. 2025;35(1):49–60. doi: 10.1007/s00330-024-10958-3 38992110

[7] Wang C, Yu J, Lu M, Li Y, Shi H, Xu Q. Diagnostic efficiency of diffusion sequences and a clinical nomogram for detecting lymph node metastases from rectal cancer. Acad Radiol. 2022;29(9):1287–95. doi: 10.1016/j.acra.2021.10.009 34802905

[8] Jia H, Jiang X, Zhang K, Shang J, Zhang Y, Fang X, et al. A nomogram of combining IVIM-DWI and MRI radiomics from the primary lesion of rectal adenocarcinoma to assess nonenlarged lymph node metastasis preoperatively. J Magn Reson Imaging. 2022;56(3):658–67. doi: 10.1002/jmri.28068 35090079

[9] Jansen JFA, Stambuk HE, Koutcher JA, Shukla-Dave A. Non-gaussian analysis of diffusion-weighted MR imaging in head and neck squamous cell carcinoma: a feasibility study. AJNR Am J Neuroradiol. 2010;31(4):741–8. doi: 10.3174/ajnr.A1919 20037133 PMC2854270

[10] Qin Y, Tang C, Hu Q, Yi J, Yin T, Ai T. Assessment of prognostic factors and molecular subtypes of breast cancer with a continuous-time random-walk MR diffusion model: using whole tumor histogram analysis. J Magn Reson Imaging. 2023;58(1):93–105. doi: 10.1002/jmri.28474 36251468

[11] Karaman MM, Zhang J, Xie KL, Zhu W, Zhou XJ. Quartile histogram assessment of glioma malignancy using high b-value diffusion MRI with a continuous-time random-walk model. NMR Biomed. 2021;34(4):e4485. doi: 10.1002/nbm.4485 33543512

[12] Brown G, Richards CJ, Bourne MW, Newcombe RG, Radcliffe AG, Dallimore NS, et al. Morphologic predictors of lymph node status in rectal cancer with use of high-spatial-resolution MR imaging with histopathologic comparison. Radiology. 2003;227(2):371–7. doi: 10.1148/radiol.2272011747 12732695

[13] Song M, Wang Q, Feng H, Wang L, Zhang Y, Liu H. Preoperative grading of rectal cancer with multiple DWI models, DWI-derived biological markers, and machine learning classifiers. Bioengineering (Basel). 2023;10(11):1298. doi: 10.3390/bioengineering10111298 38002422 PMC10669695

[14] Amornsiripanitch N, Bickelhaupt S, Shin HJ, Dang M, Rahbar H, Pinker K, et al. Diffusion-weighted MRI for unenhanced breast cancer screening. Radiology. 2019;293(3):504–20. doi: 10.1148/radiol.2019182789 31592734 PMC6884069

[15] Chang H, Wang D, Li Y, Xiang S, Yang YX, Kong P, et al. Evaluation of breast cancer malignancy, prognostic factors and molecular subtypes using a continuous-time random-walk MR diffusion model. Eur J Radiol. 2023;166:111003. doi: 10.1016/j.ejrad.2023.111003 37506477

[16] Wang W, Wu J, Shen Q, Li W, Xue K, Yang Y, et al. Assessment of pathological grade and variants of bladder cancer with a continuous-time random-walk diffusion model. Front Oncol. 2024;14:1431536. doi: 10.3389/fonc.2024.1431536 39211555 PMC11357921

[17] Gracia Bara MT, Gallardo-Higueras A, Moreno EM, Laffond E, Muñoz Bellido FJ, Martin C, et al. Hypersensitivity to gadolinium-based contrast media. Front Allergy. 2022;3:813927. doi: 10.3389/falgy.2022.813927 35386665 PMC8974732

[18] Todd DJ, Kay J. Gadolinium-induced fibrosis. Annual Review of Medicine. 2016;67:273–91. doi: 10.1146/annurev-med-063014-124936 26768242

[19] Kazama T, Takahara T, Hashimoto J. Breast cancer subtypes and quantitative magnetic resonance imaging: a systemic review. Life (Basel). 2022;12(4):490. doi: 10.3390/life12040490 35454981 PMC9028183

[20] Kim EJ, Kim SH, Park GE, Kang BJ, Song BJ, Kim YJ, et al. Histogram analysis of apparent diffusion coefficient at 3.0t: correlation with prognostic factors and subtypes of invasive ductal carcinoma. J Magn Reson Imaging. 2015;42(6):1666–78. doi: 10.1002/jmri.24934 25919239

[21] Li L, Wang K, Sun X, Wang K, Sun Y, Zhang G, et al. Parameters of dynamic contrast-enhanced MRI as imaging markers for angiogenesis and proliferation in human breast cancer. Med Sci Monit. 2015;21:376–82. doi: 10.12659/MSM.892534 25640082 PMC4324575

[22] Kim Y, Ko K, Kim D, Min C, Kim SG, Joo J, et al. Intravoxel incoherent motion diffusion-weighted MR imaging of breast cancer: association with histopathological features and subtypes. Br J Radiol. 2016;89(1063):20160140. doi: 10.1259/bjr.20160140 27197744 PMC5257327

[23] Kim SH, Cha ES, Kim HS, Kang BJ, Choi JJ, Jung JH, et al. Diffusion-weighted imaging of breast cancer: correlation of the apparent diffusion coefficient value with prognostic factors. J Magn Reson Imaging. 2009;30(3):615–20. doi: 10.1002/jmri.21884 19711411

[24] You C, Zhang Y, Chen Y, Hu X, Hu D, Wu J, et al. Evaluation of background parenchymal enhancement and histogram-based diffusion-weighted image in determining the molecular subtype of breast cancer. J Comput Assist Tomogr. 2021;45(5):711–6. doi: 10.1097/RCT.0000000000001239 34546678

[25] Ottaiano A, Santorsola M, Capuozzo M, Perri F, Circelli L, Cascella M, et al. The prognostic role of p53 mutations in metastatic colorectal cancer: a systematic review and meta-analysis. Crit Rev Oncol Hematol. 2023;186:104018. doi: 10.1016/j.critrevonc.2023.104018 37150312

[26] Wang P, Liang J, Wang Z, Hou H, Shi L, Zhou Z. The prognostic value of p53 positive in colorectal cancer: a retrospective cohort study. Tumour Biol. 2017;39(5):1010428317703651. doi: 10.1177/1010428317703651 28468582

[27] Li L, Chen W, Yan Z, Feng J, Hu S, Liu B. Comparative analysis of amide proton transfer MRI and diffusion-weighted imaging in assessing p53 and Ki-67 expression of rectal adenocarcinoma. J Magn Reson Imaging. 2020;52(5):1487–96. doi: 10.1002/jmri.27212 32524685

[28] Mehta R, Bu Y, Zhong Z, Dan G, Zhong P-S, Zhou C, et al. Characterization of breast lesions using multi-parametric diffusion MRI and machine learning. Phys Med Biol. 2023;68(8):10.1088/1361-6560/acbde0. doi: 10.1088/1361-6560/acbde0 36808921

[29] Talbot IC, Ritchie S, Leighton M, Hughes AO, Bussey HJ, Morson BC. Invasion of veins by carcinoma of rectum: method of detection, histological features and significance. Histopathology. 1981;5(2):141–63. doi: 10.1111/j.1365-2559.1981.tb01774.x 7216178

[30] Steiner D, Sultan L, Sullivan T, Liu H, Xiao X, LeClerc A, et al. Vascular invasion-associated gene expression is detectable in pre-surgical biopsies of stage I lung adenocarcinoma. Nat Commun. 2026;17(1):2581. doi: 10.1038/s41467-026-70600-2 41876493 PMC13013712

[31] Nagtegaal ID, Knijn N, Hugen N, Marshall HC, Sugihara K, Tot T, et al. Tumor deposits in colorectal cancer: improving the value of modern staging-a systematic review and meta-analysis. J Clin Oncol. 2017;35(10):1119–27. doi: 10.1200/JCO.2016.68.9091 28029327

[32] Glynne-Jones R, Wyrwicz L, Tiret E, Brown G, Rödel C, Cervantes A, et al. Rectal cancer: ESMO Clinical Practice Guidelines for diagnosis, treatment and follow-up. Ann Oncol. 2017;28(suppl_4):iv22–40. doi: 10.1093/annonc/mdx224 28881920

[33] Kim Y, Chung J-J, Yu J-S, Cho E-S, Kim JH. Preoperative evaluation of lymphovascular invasion using high-resolution pelvic magnetic resonance in patients with rectal cancer: a 2-year follow-up study. J Comput Assist Tomogr. 2013;37(4):583–8. doi: 10.1097/RCT.0b013e31828d616a 23863536

