# Supplementary material for: Investigating the potential association between the continuous-time random-walk MR diffusion model and lymph node metastasis and vascular invasion in patients with rectal cancer: A preliminary exploratory study
Source: PLoS One. 2026 Oct 30;21(10):e0361487. doi: 10.1371/journal.pone.0361487 (PMC13722178; doi:10.1371/journal.pone.0361487)
Supplement: Supplementary file 1 [file pone.0361487.s001.docx]

Supplementary Figure S1. Exploratory analysis of the association between β parameter and p53 status. With only 3 p53 wild-type cases (n=40 mutant, n=3 wild-type), this analysis is severely underpowered. The unadjusted AUC was 0.74 (p=0.018), but after Benjamini-Hochberg correction (adjusted p=0.08) and LOOCV (adjusted AUC=0.61), the association did not reach statistical significance. These findings are exploratory and require validation in larger cohorts.
